# Cook County Hospital

**Published:** 1881-05

**Authors:** 


### ARTICLE V.

### COOK COUNTY HOSPITAL.

REMOVAL OF A LOOSE CARTILAGE FROM THE KNEE-JOINT, AND USE OF ABSORBABLE DRAINAGE TUBES. Service of PROF. CHRISTIAN FENGER.

These tubes had been prepared by shaping pieces of bone into cylinders, two or three inches long, and about one-fifth inch in diameter, boring them into tubes, and destroying the mineral constituent by soaking in hydrochloric acid. After immersion in a solution of carbolic acid for a few days, they were kept in a fifteen per cent. solution of carbolic acid in oil. They are flexible, resemble brown rubber tubes, and have the property of being absorbed, as catgut ligatures, so that they may be used with permanent dressings, as this case showed.

Patient, Thos. K., æt. twenty-six, born in this country, had a sore knee for a number of years. Four months ago he was confined to his bed four days on account of that joint. He has been able to work since, but he could not stand on his feet longer than a couple of hours at a time, and he had much difficulty in walking. He was admitted to the hospital February 4. Esmarch's bandage was rolled around the leg and tied above the knee with rubber tubing. Two sprays were used, being held by assistants, but this has no special advantage over the stationary atomizers. An incision one and a half inches long was made on a line with the external border of the patella, and the hypo-external articular artery was tied. After all hæmorrhage had stopped, the synovial cavity was opened into, and the loose cartilage taken out. The synovial sac was closed with catgut sutures, and an absorbable drainage tube, described above, was left in the external wound to allow a free discharge till union had taken place, and then be absorbed, thus doing away with further dressings. The wound was now packed with ruffled pieces of gauze wrung out of a solution of carbolic acid, which were four or five inches square, and formed a layer more than two inches thick to absorb the discharges. A layer of salicylated cotton was placed over this, and bandages rolled over the whole. The limb was now placed in a well-padded splint, and was not disturbed for two weeks. There was no rise in the temperature, and there was no complication.

The loose cartilage is three-quarters inch long, two-thirds inch wide, and half inch thick, with round edges. It is half bony, or, as Prof. Fenger describes it, is "composed of a fibro-cartilaginous substance with calcareous matter in it resembling bone."

On removing the dressings, it was found that a part of the drainage tube had been absorbed, while its free extremity adhered to the gauze. The wound was healed. The patient is well, and has been discharged.

---

### PERFORATION, WITHOUT FRACTURE, OF THE FEMUR, BY A THIRTY-TWO-CALIBER BALL.—OPERATION BY PROF. C. FENGER. —THE FEMORAL VEIN LIGATURED.

M. F., male, æt. twenty-two, born in this country, was shot in the left thigh, five inches above the knee, December 16, 1880. He was removed to the hospital December 25, but the bullet could not be found, even after the wound had been enlarged and a purulent discharge had set in. After a careful examination by Dr. W. P. Verity, House Surgeon, it was found that the shaft of the femur had been perforated without fracture, an exceptional case.

February 4, 1881, Dr. C. Fenger operated. After using Esmarch's bandage an incision two inches long was made at the seat of entrance, and a sound passed through the femur, without encountering the ball. Another incision, two and a half inches long was made on the inside of the thigh, beside the femoral artery, and the ball was found imbedded in a muscle. The femoral vein had been cut during the operation and was carefully tied at both ends with catgut ligatures. A rubber drainage tube was passed through the femur, with both extremities free, and Lister's dressings were applied. The pulse beat 160 times in a minute, immediately after tying the vein, but soon returned to 100. The treatment consisted of quinine and whisky in tonic doses. The wound was dressed every third day, and the highest temperature, February 11, was 104; pulse 120. February 15, the drainage tube was removed; it was nearly fastened to the bone. Patient made a complete recovery in a few weeks.

---

ANOTHER CASE OF SHOT-WOUND.

J. D., æt. twenty-four, male, American, accidently shot himself through the left hand and thigh. Patient was anæsthetized, and the ball was probed for but could not be felt. He was admitted to the hospital March 16, the day following the accident, and on the next day his temperature was 103. The hand was dressed antiseptically and healed in the course of two weeks without any inflammation setting in. The wound in the thigh suppurated and an abscess formed in the thigh. March 23, the bullet was felt by the probe and extracted through a counter opening six inches below the point of entrance. A drainage tube was introduced, and patient is nearly well. Treatment consisted of quinine and whisky.

As these two cases show, although the bullet cannot always be felt in gunshot wounds, at first, it can be discovered after suppuration has taken place, but the antiseptic treatment is useful in moderating the latter, and insures a prompt recovery.

---

INCISED WOUND OF ABDOMEN.

J. L., æt. thirteen, Pole, had a shoemaker's knife in his pocket, and slipped, falling on his side, the blade of the knife entering his abdomen two inches above and one and a half inches to the right of the umbilicus. The omentum protruded. This was on March 16, and he was admitted to the hospital the next day. The wound was one inch long. The omentum being replaced, the wound was closed with a figure of 8 ligature, under the spray. March 20, the ligature was removed, the wound had healed without any constitutional or local disturbance.

A BOLD AND SUCCESSFUL OPERATION.

T. D., æt. twenty-three, American, was struck on the head by a piece of timber falling in an elevator-way, March 15. Admitted to the hospital the same day. Temperature 102.5, pulse 150. Patient was unconscious for a few hours, unable to speak for two days, the timber had struck him on the left parietal bone, near the longitudinal sinus, producing a large scalp wound. At first there was anæsthesia of the lower extremities, and pins were thrust into the soles of the feet without producing any sensation. Patient was also paralyzed, on the right side most, and had an ugly ecchymosis of left eye. There was no facial paralysis. Patient was delirious the first few nights. Although the external table of the skull was not fractured, the house surgeon, W. P. Verity, became convinced that the inner table must be broken, and he trephined, March 18. He removed two fragments of bone which compressed the dura-mater, and dressed the wound antiseptically. From that time the temperature and pulse became normal, and the patient is now well. There is always a greater tendency for the inner table of the skull to break, often extensively, after injuries by blows, but if the external plate is intact, the diagnosis is difficult; so much so, that surgeons, a few decades ago, did not think trephining justifiable in such cases.

---

WE HAVE finally made arrangements, at considerable expense, which will enable us to lay before the readers of THE CHICAGO MEDICAL JOURNAL AND EXAMINER, and in due season, a report of the transactions of the forthcoming International Medical Congress. We have no question but that this course will meet with the approval of the subscribers to this journal.

ANNUAL MORTALITY IN CHICAGO FOR THE PAST YEAR.—Ten thousand four hundred and fifty-two deaths within the city limits; 3,903 are referred to zymotic diseases; 53.5 per cent. were under 5 years of age.

